# Modelling Inhomogeneity of Veneer Laminates with a Finite Element Mapping Method Based on Arbitrary Grayscale Images

**DOI:** 10.3390/ma13132993

**Published:** 2020-07-05

**Authors:** David Zerbst, Christian Liebold, Thomas Gereke, André Haufe, Sebastian Clauß, Chokri Cherif

**Affiliations:** 1 Mercedes Benz AG, Research & Development, 71063 Sindelfingen, Germany; sebastian.clauss@daimler.com; 2 DYNAmore GmbH, 70565 Stuttgart, Germany; christian.liebold@dynamore.de (C.L.); andre.haufe@dynamore.de (A.H.); 3 Faculty of Mechanical Science and Engineering, Institute of Textile Machinery and High Performance Material Technology (ITM), Technische Universität Dresden, 01062 Dresden, Germany; thomas.gereke@tu-dresden.de (T.G.); chokri.cherif@tu-dresden.de (C.C.)

**Keywords:** veneer, numerical modelling, annual rings, parameter identification, grayscale mapping

## Abstract

Failure and deformation behavior of veneer laminates of ring porous wood species vary with the individual arrangement of early- and latewood zones over a veneer sheet. Therefore, a method is presented, where local failure and damage modes are considered for finite element models with respect to forming simulations, during the development process of automotive interior trim parts. Within the mapping tool Envyo, a routine has been realized for the discretization of early- and latewood zones from ash wood veneer surfaces to finite element meshes. The routine performs the following steps: reading a grayscale image of known size and generation of a point cloud based on the number of pixels; transformation and scaling of the generated point cloud to align with a target finite element mesh; nearest neighbor search and transfer of grayscale values to the target mesh element centroids; assigning part and therefore material properties to the target elements based on the mapped grayscale value and user-defined grayscale ranges. Due to the absence of measurement data for early- and latewood, optimization was used to identify locally varying material constants. A set of material input parameters for early- and latewood was created, calibrating the force-displacement response of tensile test simulations to corresponding experimental curves. The numerical results gave a very good agreement to the failure behavior of tensile tests in the loading directions longitudinal and transverse to the fiber orientation. Furthermore, in a stochastic analysis the characteristic distribution of tensile strength and ultimate strain could be verified for the suggested procedure. The introduced modelling approach can be applied for the discrete implementation of inhomogeneity to numerical simulations.

## 1. Introduction

In automotive manufacturing, veneer sheets are formed into a 3D geometry for the production of trim parts with wood surfaces. During the vehicle development process, the design of forming tools is derived based on hands-on experience of the manufacturing industry. Estimations of formability are difficult due to the environmental-dependent and highly varying material properties of wood. The present contribution introduces developments towards the prediction of the formability of veneer products in order to improve forming tool design and fixing concepts.

**Figure 1:**
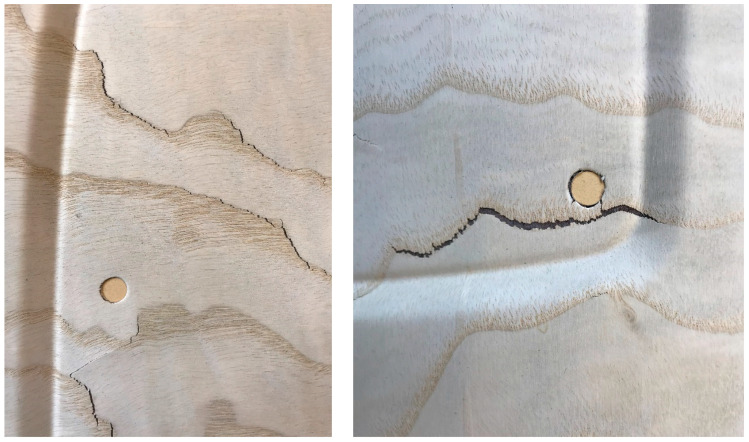
Two examples of veneer laminate sheets after forming into a trim part geometry, which show characteristic fracture along earlywood (EW) zones.

Bellair [1] presented preliminary considerations on material modeling of veneers for forming simulations. Based on extensive measurements of beech wood veneer mechanics, basic computational modelling was derived for plane material with homogenized anisotropic stiffness and strength. For the decorative trim parts in car interiors, various types of veneers such as burled, sliced, or technical veneers are used under esthetic aspects. Thereby, early- and latewood (hereafter abbreviated as EW and LW) influence the deformation and failure behavior in the forming process due to their different, locally varying properties (Figure 1). For the prediction of the formability of veneers, these characteristics have to be captured by structural mechanical modelling.

There are different ways of considering the structural inhomogeneity of wood for computational models. Most research was done in the field of civil engineering regarding the prediction of effective strength and stiffness of timber and timber products, depending on randomly varying mechanical properties [2]. Those problems require probabilistic approaches as discussed in references [3,4]. Similar methods could be mentioned for the implementation of the repetitive structure of EW and LW to veneers, analogue to reference [5]. There, imperfections in the thickness of sheet metals, caused by the manufacturing process of rolling, were taken into account for forming simulations. The uncertain geometrical parameter of thickness was distributed with a periodic function over the blank sheet. The same procedure could be applied for the material parameters of stiffness and strength to obtain varying localized failure for veneer laminate forming simulations. As opposed to stochastic approaches, discrete methods for the implementation of imperfections were applied in reference [6]. There, an algorithm is presented for the reconstruction of knot geometries in timber boards, based on laser scanning measurements of fiber angles. The implementation of the obtained morphology to computational models for the prediction of strength of timber beams and boards is shown in reference [7]. 

Various sources deal with the material modelling of microstructural related mechanics on lower length scales, summarized in reference [8]. In reference [9], a hierarchical model considering elastic stiffness of softwood is introduced. The analytical model includes mechanics from micro fibril-reinforced layers inside the cell wall on the nanoscale, multilayered cell walls on the microscale, hexagon-shaped wooden cells on the mesoscale, and the macrostructure with alternating annual rings. Lukacevic et al. [10] propose microstructural modelling based on unit cells of cell walls for EW and LW, arranged in separate layers. This allows the identification of brittle and ductile failure mechanisms on the annual ring scale and the derivation of a multisurface failure criterion in reference [11]. 

The present contribution uses a mapping method for a direct discretization of the varying structural properties of veneer sheets. Mapping is applied widely in digital prototyping within the automotive development process. It is commonly used to combine different disciplines of process and structural simulations. For example, the transfer of preliminary damage induced through sheet metal forming simulations to a crash simulation [12] or for other fiber reinforced plastic materials, such as in reference [13].

In this work, the new method of grayscale mapping is introduced. It is applied to assign EW and LW zones of ash wood veneer laminates to meshes of finite shell elements. Tensile tests are used to obtain material constants of EW and LW through optimization. The presented modelling approach allows for the prediction of failure due to the individual structure of a veneer sheet.

## 2. Methods

### 2.1. Experimental Database

#### 2.1.1. Material Composition and Testing Methods

**Figure 2:**
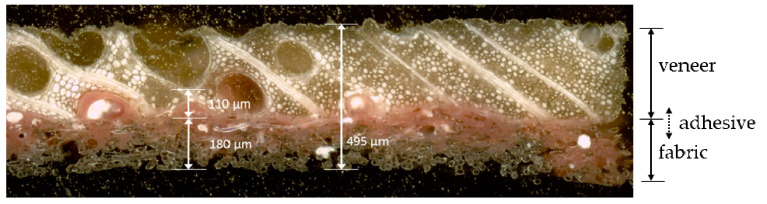
Layers of veneer laminate (VL) in a transverse cross section (2–3 plane).

Preliminary analysis of the material failure behavior was done under tensile loading, as a database for the model calibration and validation. The analyzed material was a laminate of veneer and a nonwoven fabric bonded with a phenolic resin (veneer laminate, abbr.: VL) (Figure 2). To reduce the number of tests, the VL was analyzed in the composite form. Two VL sheets were produced, for the preparation of tensile samples. The orientation of the fabric support layer was in the direction perpendicular to the fiber direction of veneer. The wood species was ash wood, which is frequently used for decorative surfaces in automotive interiors. The veneer sheets were taken from the same stack, right on top of each other. That way the two sheets had almost identical properties. Ten samples were tested in each case for the directions longitudinal and transverse to the fiber direction. The specimens were immersed in water before testing to obtain a material behavior closer to conditions in the veneer forming process. Immersion was done at room temperature for one hour until constant mass was reached. The procedure in detail and a full characterization of the material is depicted in reference [14]. Tensile tests were carried out with a standard testing device (Inspekt 10, Hegewald & Peschke, Nossen, Germany). Testing was done according to reference [15] on specimens of 120 × 10 mm^2^. Thereby, the area of investigation between the clamping jaws was 80 × 10 mm^2^. 

#### 2.1.2. Tensile Properties

**Figure 3:**
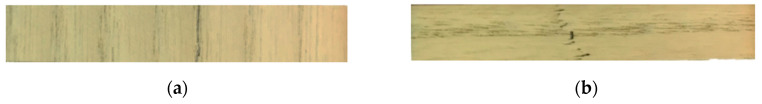
Perpendicular (**a**) and longitudinal tensile samples (**b**) of VL with failure.

The results of the tensile tests showed anisotropic behavior in Young’s modulus, tensile strength and ultimate strain (Table 1). For a ring porous wood species like ash wood the structural difference between EW and LW is strongly developed. Due to significantly lower density, the EW zone is much weaker compared to LW. Thus, rupture in the transverse direction occurs exclusively in an EW zone (Figure 3a). Figure 2 explains the structural reason for this effect. In some cases, the lumens of EW vessels have such a large diameter that the cohesion of the material is almost exclusively given by the fabric support and the adhesive, which is much more homogeneous in its structural organization. Consequently, the variation of transverse strength was relatively low compared to the longitudinal strength (Table 1). The same relationship was observed for the variation of ultimate strain values. Ultimate strain in the longitudinal direction was found to have a higher variation. A longitudinal tensile sample of the presented series contained one or two EW zones. Thus, the response in strength and strain under longitudinal tension was more dependent on the individual, microstructural related constitution of a single EW or LW zone. Otherwise, the measured values in the transverse direction depended on the tensile response of several EW and LW zones and, consequently, properties were more homogeneous with lower variation. 

### 2.2. Material Model

**Figure 4:**
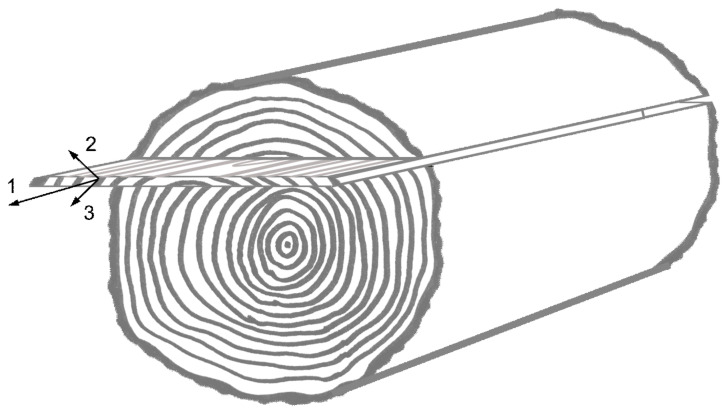
Schematic view of a veneer sheet sliced from a trunk with orthotropic material directions.

Following the experimental analysis, considerations on modelling were applied for the composite material, although there are individual influences of the separate layers on the overall mechanics. The VL compound is dominated by the mechanical properties of the veneer layer. Wood is commonly considered to be orthotropic, e.g., in reference [16], where the three growth related material directions are defined in the longitudinal (1) fiber direction, parallel to the trunk axis, as well as in the radial (2) and tangential (3) directions of the growth rings (Figure 4). 

For veneers, the annual ring angle in the 2–3 plane differs with the location from where a sheet was sliced from the trunk. This angle is hard to differentiate for thin layers. Thus, the VL is assumed to be a transversely isotropic material with uniaxial arranged fibers. Because of the planar spatial extent of VLs, plane stress is assumed to be adequate for modelling. Hence, failure occurs due to fiber-stresses acting parallel ($\sigma_{11}$) or inter-fiber stresses acting normal ($\sigma_{22}$) and in shear direction ($\sigma_{12}$), respectively. Analogue considerations on failure criteria were made for fiber reinforced composite materials, e.g., in reference [17]. Finite element (FE) analysis was carried out with LS-DYNA, which has a library with various material models dedicated for composite modeling. In this work, the material model *MAT_LAMINATED_COMPOSITE_FABRIC (MAT_058) was chosen for VL modeling, assuming comparable characteristics of fabrics and the VL structure.

**Figure 5:**
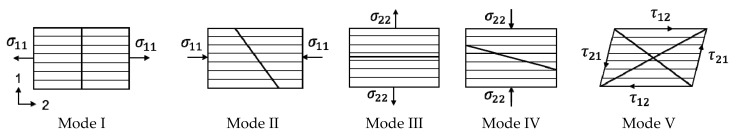
Plane stress failure criteria of VL from Equations (1)–(5).

Different works deal with the failure modelling of wood. Multisurface criteria were introduced with high accuracy for orthotropic failure of clear wood [11,15,18]. Furthermore, some general criteria such as the Tsai-Wu hypothesis were applicable with sufficient estimations of failure [19]. Due to the limited number of measurements with combined stresses such as biaxial tests, a simple maximum stress criterion is applied for the modelling of VL. Direction dependent stress components are uncoupled, and failure is assumed whenever material strength in longitudinal (X) or transverse (Y) tension (subscript T), compression (subscript C) or shear ($S_{C}$) is reached. Consequently, five failure modes with the following criteria $e_{1/2,C/T}^{2}$ apply to VL (Figure 5). Fracture in the respective mode is identified if the corresponding value reaches 0.

Mode I: parallel tensile failure $\sigma_{11}>0$

(1) $$e_{1,T}^{2}={\left(\frac{\sigma_{11}}{X_{T}}\right)}^{2}-1$$

Mode II: parallel compressive failure $\sigma_{11}<0$

(2) $$e_{1,C}^{2}={\left(\frac{\sigma_{11}}{X_{C}}\right)}^{2}-1$$

Mode III: transverse tensile failure $\sigma_{22}>0$

(3) $$e_{2,T}^{2}={\left(\frac{\sigma_{22}}{Y_{T}}\right)}^{2}-1$$

Mode IV: transverse compressive failure $\sigma_{22}<0$

(4) $$e_{2,C}^{2}={\left(\frac{\sigma_{22}}{Y_{C}}\right)}^{2}-1$$

Mode V: Shear failure ($\left|\tau_{12}\right|>0$)

(5) $$e_{12,S}^{2}={\left(\frac{\left|\tau_{12}\right|}{S_{C}}\right)}^{2}-1$$

The stress-strain behavior of VL is nonlinear, especially under loading in the transverse direction. Nonlinear stiffness was implemented using classical continuum damage mechanics according to reference [20]. Nominal stresses according to Hooke’s law are replaced by effective stresses $\hat{\sigma }$. Within this interpretation, the constitutive tensor C is a function of the damage variable $\omega_{ij}$, which takes values between 0 (fully elastic material) and 1 (fully damaged material).

(6) $$\hat{\sigma }=C\left(\omega_{ij}\right)\varepsilon$$

(7) $$C\left(\omega_{ij}\right)=\frac{1}{D}\left[\begin{array}{ccc} \left(1-\omega_{11}\right)E_{11} & \left(1-\omega_{11}\right)\left(1-\omega_{22}\right)\nu_{21}E_{22} & 0\\ \left(1-\omega_{11}\right)\left(1-\omega_{22}\right)\nu_{12}E_{11} & \left(1-\omega_{22}\right)E_{22} & 0\\ 0 & 0 & D\left(1-\omega_{12}\right)G_{12} \end{array}\right]$$

*D* is given as:

(8) $$D=1-\left(1-\omega_{11}\right)\left(1-\omega_{22}\right)\nu_{12}\nu_{21}>0$$

Analogue to the failure modes of Equations (1)–(5), $\omega_{11}$ and $\omega_{22}$ have different values in tension and compression:

$$\omega_{ij}=\omega_{ij,C}\;\mathrm{if}\;\sigma_{ij}<0;\;\omega_{ij}=\omega_{ij,T}\;\mathrm{if}\;\sigma_{ij}>0$$

Damage evolves exponentially with the following relations:

(9) $$\omega_{11C,T}=1-\exp\left[-\frac{1}{m_{11C,T}\;e}{\left(\frac{E_{11}\varepsilon_{11}}{X_{C,T}}\right)}^{m_{11C,T}}\right]$$

(10) $$\omega_{22C,T}=1-\exp\left[-\frac{1}{m_{22C,T}\;e}{\left(\frac{E_{22}\varepsilon_{22}}{Y_{C,T}}\right)}^{m_{22C,T}}\right]$$

(11) $$\omega_{12S}=1-\exp\left[-\frac{1}{m_{12S}\;e}{\left(\frac{G_{12}\varepsilon_{12}}{S_{C}}\right)}^{m_{12S}}\right]$$

with:

(12) $$m_{11C,T}=\frac{1}{\ln\left(\varepsilon_{11C,T}\frac{E_{11}}{X_{C,T}}\right)}$$

(13) $$m_{22C,T}=\frac{1}{\ln\left(\varepsilon_{22C,T}\frac{E_{22}}{Y_{C,T}}\right)}$$

(14) $$m_{12S}=\frac{1}{\ln\left(\varepsilon_{12S}\frac{G_{12}}{S_{C}}\right)}$$

The variable *e* in the denominator of Equations (9)–(11) represents the Euler number, i.e., *e* = 2.71828, and should not be confused with the failure variables defined in Equations (1)–(5). The strength values of Equations (1)–(5) act as a threshold value for the material failure. Young’s moduli $E_{ij}$ and a strain value $\varepsilon_{ij,C,T}$ at maximum strength adjust the shape of the stress-strain curve with initial gradient and final elongation. Element erosion is implemented with an effective strain criterion between 0 and 1 (meaning 0% resp. 100% strain), which is usually set relatively high if anisotropic failure is active.

The VL material fails with a sudden crack under tensile loading. Here, failure is triggered with the strength parameters $X_{T}$ and $Y_{T}$. Under compressive and shear loading, the material behaves similar to honeycomb structures with a continuous crushing and buckling of fiber lumens. To consider this physical behavior, MAT_058 provides the option of keeping perfect plasticity after reaching the strength limits $X_{C}$, $Y_{C}$ and $S_{C}$ by setting the stress limit factor $SLIM_{C}$ to 1 (see [21]). In the forming process of automotive interior surfaces, failure under compression has to be evaluated until this crushing reaches unacceptable magnitudes under esthetic criteria. Those limits have to be defined in hardware forming tests and were not examined more closely within this contribution.

### 2.3. Modelling Strategy by Grayscale Mapping

**Figure 6:**
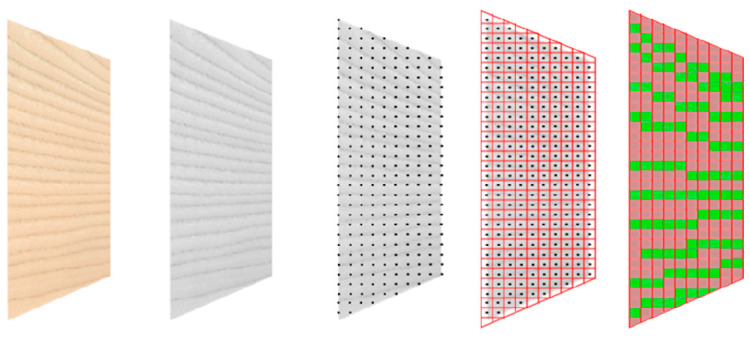
Processing steps of the mapping procedure from left to right: source image in raw format, export to portable gray map (*pgm) format, point cloud generation, alignment with target finite element mesh, allocation of part IDs.

The high dependency of material failure of the local varying structure, observed in Section 2.1.2, requires material modelling based on the annual ring scale. Therefore, a straightforward discretization method is introduced to assign the differences in mechanical behavior of EW and LW to corresponding spatially discretized finite element (FE) meshes. Wider vessels and thinner cell walls in the EW zones contrast from LW in a darker color as may be observed by the naked eye (Figure 6). Based on these observations an image-processing algorithm was implemented into the mapping tool Envyo. With this new feature, brighter and darker structures could be distinguished based on their gray values. The method of grayscale mapping was first mentioned in reference [22] and will be abbreviated as GSM in the following.

**Figure 7:**
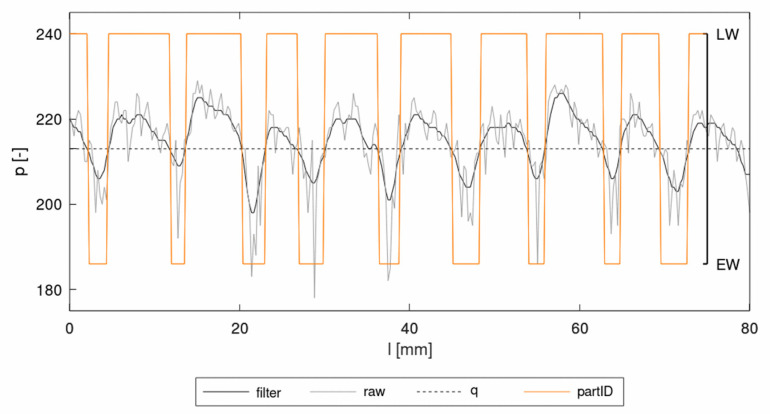
Gray value *p* from a single pixel row over the length of a tensile sample.

The first step of the procedure was the recording of an image of the wood surface as a database for the simulations. The resulting colored image was then transferred into an ASCII-readable representation of a grayscale image using freely available software GIMP (Gnu Image Manipulation Program). The result was exported as an ASCII-based *pgm (portable gray map) format, which holds information about the number of pixels in the vertical and horizontal directions as well as the grayscale value for each pixel within the range of 0 (black) and 255 (white). In order to avoid the mapping of sudden single peaks and artifacts, a default Gaussian filter was applied. The filter smoothed sharp increases of the gray value. With this treatment the alteration of EW and LW contrasted more clearly (Figure 7). The software Envyo read the resulting *pgm file and calculated the positions of the pixels using the information about the number of pixels in vertical and horizontal direction and the information provided by the user about the image size. In some cases, this generated point cloud had to be aligned with the target FE mesh before a proper data transfer could be performed. To do this, several standard transformation procedures such as scaling, rotation and translation were available. Once the point cloud and the mesh were aligned, a bucket sort and search algorithm was used to detect the nearest points (or pixel) of the point cloud to the centroid of the target mesh. This search was started from the target mesh and a bucket sort algorithm based on the average element length helped to speed up the search process. The grayscale values of the pixel closest to the element center were transferred to the target element midpoint and stored there for further processing. It should be emphasized that in the current version, no averaging of multiple grayscale values within a certain area was performed and that all elements, regardless of their distance to the nearest grayscale pixel would be assigned a grayscale value. Within the LS-DYNA nomenclature, elements were assigned to “parts” where material cards could be equipped. Consequently, different parts would be created, based on multiple grayscale ranges, which would be assigned to a part ID, which could then hold different material properties considering the properties of EW and LW. Thus, gray values of the smoothed *pgm file were allocated to part IDs for EW or LW in ranges of 255 ≥ q > 0, where q was the threshold value (Figure 7). 

**Figure 8:**
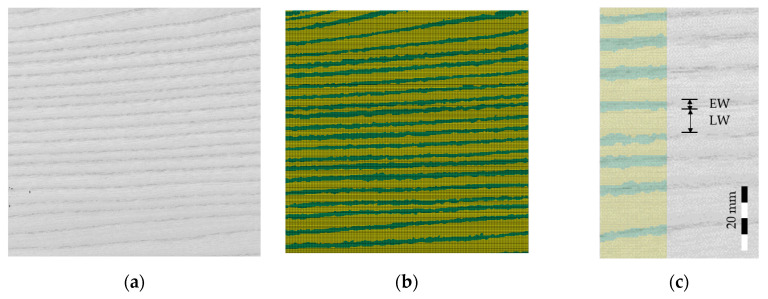
(**a**) Original *pgm image for mapping (**b**) finite element (FE) mesh with allocation of EW (green) and LW (yellow) with q = 213. (**c**) Borders of EW and LW on mesh and image.

A single VL sheet of the test material with dimensions of 200 × 200 mm^2^ was used for the analysis. The image was taken with a conventional flatbed scanner in JPG format. Target meshes were prepared with dimensions of 200 × 200 mm^2^, with constant element size of 0.5 × 0.5 mm^2^. Different grayscale values q were compared, to find a good agreement for the mapping configuration with the visual wood structure. The parameter q should be adjusted to create EW and LW parts in coincidence with the anatomical borders of the annual rings. This was given for q = 213, under the condition of the resolution given through the element length (Figure 8). With these ranges the ratio between EW and LW was 1:2.12 and the average EW width was ~3 mm. Nevertheless, the capture of the annual ring structure could only be done visually on the macroscopic level and the borders between EW and LW were not always sharply delineated on the images. Consequently, a proper calibration of material parameters had to be applied, in addition to the mapping idea.

### 2.4. Identification of Local Material Parameters

The basic concept of the GSM approach is the assumption of two different materials for EW and LW as opposed to a homogenized model with averaged properties. Measurements of local material constants are tedious, especially under elevated moisture and temperature, and require comprehensive technical equipment and digital image correlation systems [23]. In order to define material properties for the proposed modelling approach of two materials a consistent handling of material parameters has to be found. Thus, a straightforward strategy was applied using reverse engineering methods. 

**Figure 9:**
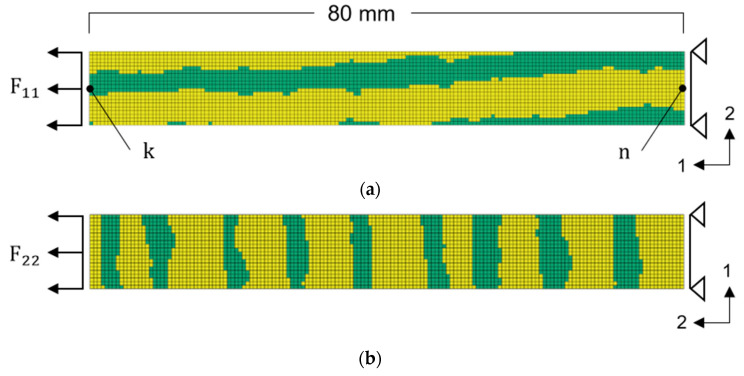
(**a**) FE model of a tensile sample with fibers oriented in the longitudinal direction. (**b**) FE model of a tensile sample with fibers oriented in the transverse direction.

The global stress-strain response of a VL sample under loading conditions is the combination of the stiffer LW zones and the EW zones of lower stiffness and strength. Based on these considerations, material parameters were defined as variables for EW and LW. In iterative simulation runs, those parameters were adjusted to fit the model response to force-displacement curves from the tensile tests of Section 2.1.2, in order to find an optimum combination of EW and LW parameters within the assumed ranges. The curve matching was done by optimization with LS-OPT. The optimization setup was prepared as follows: A model of a tensile sample with the dimensions of 80 × 10 mm^2^ was created with constant element size of 0.5 × 0.5 mm^2^, which corresponded to the smallest element size used for forming simulations of decorative trim parts. With an element length of 0.5 mm typical radii of trim part geometries were meshed properly, regarding stable contacts and deformations. Using this model as target mesh, EW and LW parts were created by GSM with the source image of Figure 8a, one for the longitudinal and one for the transverse direction. Therefore, the mapped region, which was transferred to the models, was chosen randomly, but in a way such that the FE tensile sample appeared to have an average distribution of EW and LW (Figure 9). Material directions were rotated on the elements according to the global fiber direction. 

The simulation of the tensile test was realized by fixing the bottom edge nodes and applying a prescribed translational displacement to the upper edge nodes. One force-displacement curve for each direction was chosen for the calibration from the experiments (Section 2.1.2). Force and displacement values of the selected curves were close to the mean values of the test series. To match simulation and experimental force-displacement curves, force output from the constraint bottom nodes was used. Displacement was derived through the distance between node k and the reference node *n* (Figure 9). The optimization task may be written as:

(15) $$\mathrm{MSE}\left(\omega_{ij}\right)=\frac{1}{P}\sum_{m=1}^{n}{\left\|G_{m}\left(\omega_{ij}\right)-H_{m}\right\|}^{2}\to \min$$

The objective was to minimize the distance between the simulation response $G_{m}$ as function of the nominated parameters $\omega_{ij}$ and $H_{m}$ containing the experimental force-displacement points. Optimization was done by minimization of the mean squared error MSE over the range of load stages from *m* = 1 to *n* = 100.

Constituent damage parameters according to Equations (9)–(14) were defined as variables with ranges specified based on the preliminary, experimental observations (see Section 2.1.2). That way, LW was assumed to have higher Youngs’s moduli $E_{ij}$, higher strength $X_{T}$ or $Y_{T}$, and lower ultimate strain $\varepsilon_{ij,T}$ relative to global values derived from the measurements (Table 1 and Table 2). Ranges of EW parameters, in turn, were specified with lower $E_{ij}$ and $X_{T}$ or $Y_{T}$, but higher $\varepsilon_{ij,T}$. There was only a small range specified for $Y_{T,EW}$ because the EW zone is acting like a predetermined failure zone and thus EW strength coincides with the global strength. Poisson’s ratio $\nu_{12}$ was kept constant for both EW and LW according to measurements of reference [14], whereas $\nu_{21}$ was adjusted automatically keeping the symmetry condition of the stiffness tensor:

(16) $$\nu_{21}=\left(\frac{E_{22}}{E_{11}}\right)\cdot \nu_{12}$$

Element erosion was triggered once the effective strain of 0.6 was reached.

Within the optimization setup, the d-optimal algorithm was chosen to find optimal combinations of the continuous parameters. Following the d-optimal default settings, 10 designs with individual combinations of the parameters were tested in implicit simulations of the tensile test for each iteration step. The design space was reduced after each iteration based on metamodel approximations. A total of 15 iterations were defined as termination criterion. With that procedure, the separate material input of EW and LW zones was fully calibrated.

## 3. Results

### 3.1. Model Calibration

**Figure 10:**
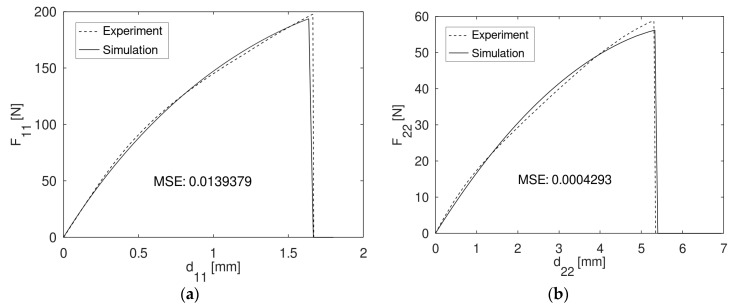
(**a**) Force-displacement curve of experiment and simulation, in the longitudinal direction. (**b**) Force-displacement curve of experiment and simulation, in the transverse direction.

As explained earlier, the calibration of EW and LW parameters was based on assumptions. The decision, which parameters were specified as variables and in which ranges they were moving, was made without restrictions with respect to the solution space. However, the calibration on global force-displacement curves provided very good results for both directions, after reaching the termination criteria of 15 iterations. The longitudinal experimental curve and simulation curve matched with a mean square error of 0.0139 (Figure 10a). In the transverse direction, a mean square error of 0.0004 was achieved (Figure 10b), which justifies the usage of *MAT_058.

**Figure 11:**
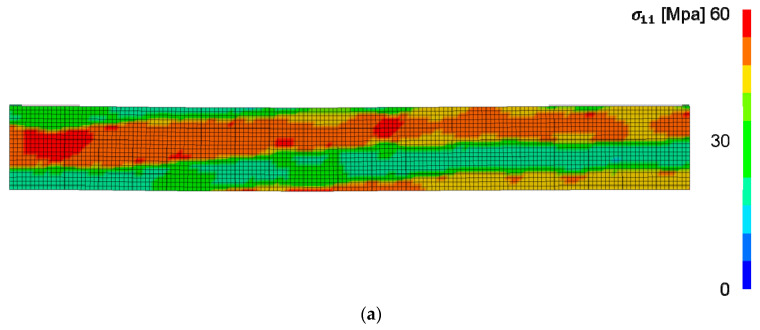

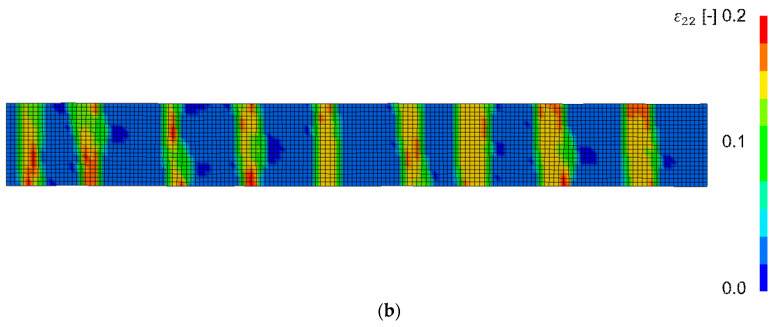
(**a**) Stress distribution at plot state before failure in the longitudinal direction. (**b**) Strain distribution at plot state before failure in the transverse direction.

To the knowledge of the authors, there is no sound experimental data of EW and LW constitutive properties of ring porous wood species available in the open literature to compare the numerically obtained constants. The identified parameters (Table 3) are consistent under the model assumption of two clearly differentiated materials and depend on the mapping parameter q, the element size and the defined variable ranges. Anatomically, there is a smooth transition between EW and LW. Thus, the absolute values may be unphysical. However, the global stress-strain response of the FE tensile samples is matching to the experiments with high accuracy. Thereby, the results of the simulations showed the characteristic deformation and fracture behavior of VL or veneers under tension in general. With load direction parallel to the fibers, the sample failed in the transverse fracture plane in EW and LW at the same time. Under a prescribed displacement from the boundary condition, the LW zones took higher stresses due to higher stiffness (Figure 11a). The results in the transverse direction showed the characteristic strain distribution with higher strain concentration in the EW zones, which was observed as well in reference [14] (Figure 11b). Failure occurred in one EW zone as expected. 

### 3.2. Stochastic Analysis

The failure behavior of the presented modelling approach was analyzed in a stochastic study. Ten FE tensile samples for the longitudinal and the transverse direction were created by GSM from different regions of the source image. Thereby the image was shifted randomly, using the translational displacement options in the Envyo input deck. The calibrated material cards for EW and LW were used for the simulations. 

**Figure 12:**
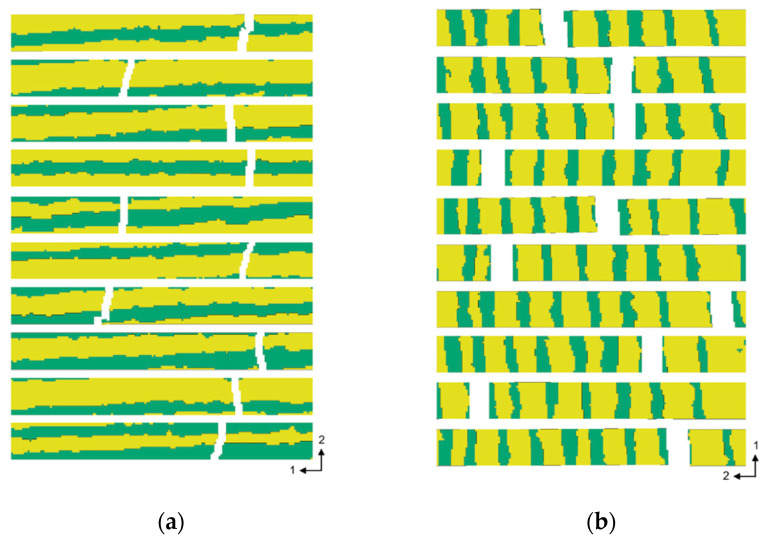
FE models of tensile samples of the first plot state after failure in (**a**) the longitudinal direction and (**b**) the transverse direction.

All simulation samples showed the characteristic failure behavior as known from material testing. The exact point of failure in both directions localized due to the geometrical distribution of EW and LW originating from the mapping process. The longitudinal samples failed at cross sections with higher amounts of EW (Figure 12a), which were obviously the weakest areas. In the transverse direction fracture occurred in an EW zone for all samples (Figure 12b).

**Figure 13:**
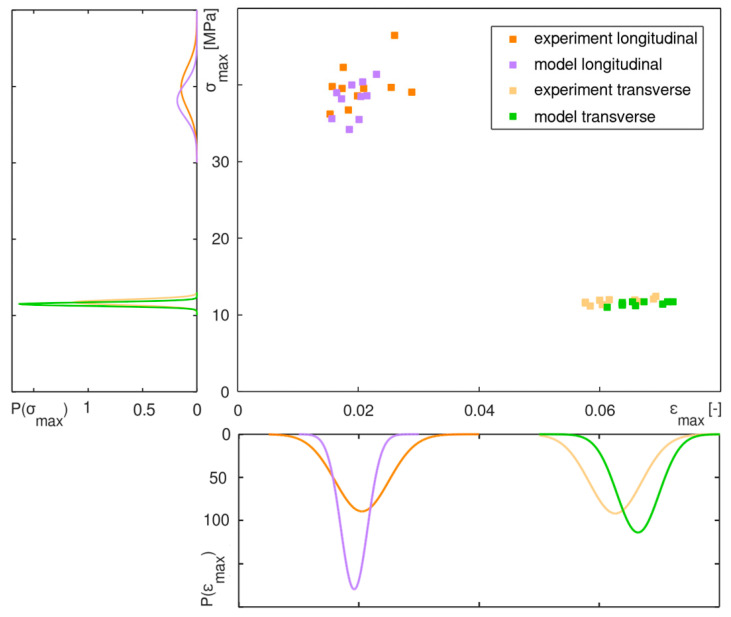
Distribution of the results of experimental and numerical strength and ultimate strain as scatter plot and in terms of the probability density function.

The distribution of the tensile strength and the ultimate strain of the models was compared to the distribution of the experimental values. The results were assumed to be normally distributed and are depicted in Figure 13 by the probability density function *P*(*x*).

(17) $$P\left(x\right)=\frac{1}{\sigma_{x}\sqrt{2\pi }}\exp\left[-\frac{1}{2}{\left(\frac{x-\mu }{\sigma_{x}}\right)}^{2}\right],$$

where *x* is the random variable, μ is the mean value and $\sigma_{x}^{2}$ is the variance.

In general, there was the same characteristic distribution of the numerical and the experimental results (Figure 13). The variation of the transverse strength was very low because it was always related to one specific EW zone for the simulations as well as for the experiments. On the other hand, the longitudinal strength was widely distributed. Here, structural conditions over a fractured cross section differed strongly with the amount of EW and LW (Figure 12a), as already mentioned in Section 2.1.2.

General differences in the mean values and the variance of the experimental and the numerical results may be caused by a different sample collection. All specimens for the experiments were cut from two sheets with almost similar properties, whereas all FE models were mapped from the source image of one of these sheets. Furthermore, they were collected from random regions on these sheets.

In general, the variance of the numerical results was lower for all series. Failure properties of the natural VL material are not only dependent on the geometrical appearance of EW and LW. They also vary with microstructural conditions on the cell wall level, e.g., the micro-fibril-angle, or even with the degree of crystallinity in the ultrastructure. Additionally, with the modelling approach the material is considered to be transversely isotropic. Therefore, the radial and the tangential plane is smeared which typically causes lower distributions. 

## 4. Summary and Conclusions

The individual structures of a veneer surface, created by sliced annual rings, were mapped to finite element meshes. Gray values could be allocated easily from images to part IDs for EW and LW. The differentiation based on a grayscale threshold value is only valid with the subsequent calibration procedure. The parameters obtained by optimization depend on the assumptions of variable ranges and fixed parameters. Thus, values of EW and LW might not correspond to experimental measurements in absolute terms. However, with this procedure the system provides the same response as global tensile tests. That way, characteristic fracture on the annual ring scale could be implemented. 

In a stochastic study of tensile test simulations of ten mapped samples, for loading directions parallel and transverse to the fiber direction, the distribution of strength and ultimate strain was analyzed. The numerical analysis showed the dependency of the variation of deformation and failure properties due to local inhomogeneity of EW and LW, for ring porous wood species. The simulation results were in coincidence with the distribution of experimental tensile tests. Finally, it can be concluded that the stress and strain fields, as well as the material failure of VL, are captured more accurately with the introduced modelling approach. The procedure can be applied in prospective forming simulations to predict the formability of a wood surface on a trim part geometry in the automotive development process.

Nevertheless, the assignment of EW and LW parts based on one static threshold value has to be used carefully. The method is sensitive for variations in color, which might occur over a veneer sheet. Thus, an absolute threshold value could lead to artifacts with the allocation of EW and LW for larger dimensions of VL. Possible ways to avoid those artifacts could be to level brightness by image manipulation or an alternative algorithm for capturing the width of EW peaks. 

Apart from the model assumption of two different materials for EW and LW, the mechanical properties of veneers vary with the density by nature. In order to introduce some generalization of the method, further improvements could be made by density mapping. The mapping input data could be obtained from a picture of a veneer surface with a light source behind it. This image of light shining through thin veneers would be a direct function of the density distribution and dependent mechanical parameters. With this source data, the absolute material constants could be respected for FE models in coincidence with an anatomically correct transition between EW and LW.

For the general material description, a material model from the LS-DYNA library was used, with formulations of transversely isotropic failure and damage. Thereby, the chosen failure criterion was well-suited for the demonstration of local failure under one-dimensional loading. Nevertheless, it has to be noted, that stresses will be overestimated under biaxial loading. In 3D forming simulations with combined stress situations a failure hypothesis with coupled stress components has to be implemented.

The adaption of the method has been made using an element size, which is typical for automotive process simulations. When applying the method to other problems, e.g., a crash simulation, the calibration of material parameters should be repeated for the individual mesh and mapping configuration. Further refinement of the mesh density was not analyzed, regarding a converged solution of the identified parameters for general usage. The presented study is one step for the automation of the process chain in digital automotive development.

Many applications of GSM are conceivable besides forming simulations of VLs. Images of veneer surfaces exist from preliminary production steps of veneers for quality grading and sorting. An estimation of effective failure properties based on those images in early manufacturing would be possible. The GSM could also be used to discretize other structural imperfections such as knots. Furthermore, the tool could map the input of more advanced imaging technics to identify inhomogeneity due to decay or moisture related defects, as mentioned in reference [24], for structural analysis of damage of timber in buildings. Generally, with this automatic discretization method all visible structural distinctions can be captured for FE simulations.

## Figures and Tables

**Table 1:** Results of tensile tests for Young’s modulus ($E_{ij}$), strength ($\sigma_{ij,max})$ and ultimate strain ($\varepsilon_{ij,max}$) given in terms of: mean value (coefficient of variance).

Loading Direction	$E_{ij}$	$\sigma_{ij,max}$	$\varepsilon_{ij,max}$
*ij*	[MPa]	[MPa]	[-]
11	3530 (11%)	40 (7%)	0.02 (22%)
22	380 (7%)	12 (3%)	0.06 (7%)

**Table 2:** Parameter set with ranges for the calibration of EW and LW material cards.

Part	Parameter	Type	Unit	Range
LW	$E_{11}$	Variable	MPa	3000–5000
	$E_{22}$	Constant	MPa	600
	$\nu_{12}$	Constant	-	0.42
	$\nu_{21}$	Dependent	-	-
	$\varepsilon_{11,T}$	Variable	-	0.01–0.04
	$\varepsilon_{22,T}$	Variable	-	0.08–0.15
	XT	Variable	MPa	40–70
	YT	Variable	MPa	20–40
EW	$E_{11}$	Constant	MPa	2000
	$E_{22}$	Variable	MPa	100–400
	$\nu_{12}$	Constant	-	0.42
	$\nu_{21}$	Dependent	-	-
	$\varepsilon_{11,T}$	Variable	-	0.03–0.08
	$\varepsilon_{22,T}$	Variable	-	0.15–0.3
	XT	Variable	MPa	25–40
	YT	Variable	MPa	10–14

**Table 3:** Calibrated parameter set of tensile properties for EW and LW.

Part	$E_{11}$	$E_{22}$	$X_{T}$	$Y_{T}$	$\varepsilon_{11,T}$	$\varepsilon_{22,T}$	$\nu_{12}$	$\nu_{21}$
	[MPa]	[MPa]	[MPa]	[MPa]	[-]	[-]	[-]	[-]
LW	4452	600	61	27	0.039	0.116	0.42	0.05660
EW	2000	136	30	12	0.056	0.207	0.42	0.02856
